# Association Between FT4/FT3 Ratio and Short‐Term Treatment Response in Differentiated Thyroid Cancer Patients

**DOI:** 10.1155/ije/7915829

**Published:** 2025-12-08

**Authors:** Sen Zhang, Shuli Niu, Ling Zhou, Yuhao Liu, Mao Deng

**Affiliations:** ^1^ Department of Nuclear Medicine, Deyang People’s Hospital, Deyang, China

**Keywords:** differentiated thyroid cancer, excellent response, FT4/FT3 ratio, prognosis prediction, thyroid hormones

## Abstract

**Background:**

Thyroid cancer is the most common endocrine tumor, with its incidence increasing worldwide. Although differentiated thyroid cancer (DTC) generally has a good prognosis, some patients still experience recurrence or persistent lesions after initial treatment. This study aimed to explore the relationship between free thyroxine (FT4)/free triiodothyronine (FT3) ratio and short‐term treatment response in patients with DTC.

**Methods:**

A retrospective analysis was conducted on 225 patients with DTC who received treatment at the Nuclear Medicine Department of Deyang People’s Hospital from 2019 to 2022. All patients underwent total or subtotal thyroidectomy followed by radioactive iodine therapy. Clinical data and preoperative laboratory test results were collected, and multivariable logistic regression analysis was used to evaluate the association between the FT4/FT3 ratio and the short‐term treatment response in DTC, as well as trend analysis and restricted cubic spline (RCS) evaluation for nonlinear relationships.

**Results:**

Among the 225 patients, 159 (70.7%) achieved an excellent response (ER). Compared to the non‐ER group, the ER group had higher levels of FT4 (*p* = 0.040). After multivariate adjustment, the FT4/FT3 ratio was significantly associated with DTC prognosis (OR = 0.40, 95% CI: 0.22–0.68). Trend analysis showed that patients in the highest quartile of FT4/FT3 ratio were more likely to achieve ER status (OR = 0.20, 95% CI: 0.07–0.55; P for trend = 0.006). RCS analysis indicated a dose–response relationship between FT4/FT3 ratio and short‐term prognosis of DTC (P for overall = 0.006; P for nonlinear = 0.887).

**Conclusion:**

The FT4/FT3 ratio is significantly associated with short‐term treatment response in patients with DTC. A higher FT4/FT3 ratio may be an independent predictive factor for a favorable prognosis in DTC patients. Future studies should further investigate its mechanisms and potential clinical applications.

## 1. Introduction

Thyroid cancer is the most common endocrine tumor [1]. Over the past 20 years, the global incidence of thyroid cancer has risen significantly, currently ranking seventh among all cancers and fifth in women [2, 3]. Notably, Rahib et al. [4] projected that by 2030, it may become the fourth most diagnosed cancer, surpassing colorectal cancer. According to data released by the China National Cancer Center in 2015, thyroid cancer ranks seventh among all malignant tumors and fourth among female malignancies [5]. Differentiated thyroid carcinoma (DTC) is the most commonly diagnosed type, and with standardized treatment, the majority of patients have a good prognosis and can achieve clinical remission. Although DTC has a relatively low overall mortality rate, it is still on an upward trend. Compared to a 5‐year survival rate of 98.7% in developed countries, such as the United States, China’s DTC has a lower 5‐year survival rate at only 84.3% [5].

Although the mortality rate of DTC is relatively low, up to 30% of patients experience recurrence or persistent lesions after initial treatment [6]. The 2015 American Thyroid Association (ATA) guidelines classified treatment response into different categories [7], with excellent response (ER) being the most favorable. Approximately 15%–20% of patients with indeterminate response (IDR) progress to structural incomplete response (SIR), while 20% of those with biochemical incomplete response (BIR) also transition to SIR. Patients in SIR have a high distant metastasis rate of up to 50%, and locally advanced patients have a specific mortality rate as high as 11% [8, 9]. Therefore, determining whether a patient is in an ER state is crucial for clinical outcomes and identifying independent factors that influence ER and implementing intervention measures may effectively improve the 5‐year survival rate.

The impact of thyroid‐stimulating hormone (TSH) on the prognosis of thyroid cancer has been extensively studied, and even when thyroid function is within the normal range, low levels of TSH may be associated with the incidence of thyroid cancer [10]. However, in thyroid nodules, low levels of TSH appear to be a protective factor for thyroid cancer [11, 12]. Thyroid hormones are synthesized and secreted by the thyroid gland, mainly thyroxine (T4) and triiodothyronine (T3), which play important roles in tumor proliferation, apoptosis, and angiogenesis. The active forms of T4 and T3 are free thyroxine (FT4) and free triiodothyronine (FT3), respectively, and they can only exert physiological effects after entering cells. The physiological activity of FT3 is stronger than that of FT4, and FT4 is converted into FT3 by deiodinase in peripheral tissues. Therefore, the FT4/FT3 ratio can reflect both the activity of deiodinase in peripheral tissues and the sensitivity to thyroid hormones [13]. As an important hormone affecting the internal environment of the human body, thyroid hormones have significant effects on several tumors. Research by Nisman B et al. suggests that FT4 and the FT3/FT4 ratio play a role in the malignant transformation of breast cancer [14]. In pancreatic cancer, the conversion rate of FT4 to FT3 may be an independent factor affecting prognosis [15]. In the field of thyroid cancer, research by Sasson M et al. believes that an FT4/FT3 ratio > 3.3 increases the risk of malignant transformation of thyroid nodules by 3.6 times [16]. However, there is currently little research on whether the FT4/FT3 ratio affects short‐term ER in patients with DTC. Therefore, we conducted this retrospective study to explore the relationship between the FT4/FT3 ratio and short‐term ER in patients with DTC.

## 2. 2. Patients and Methods

### 2.1. Patients

This study retrospectively analyzed patients with DTC who were treated in the Department of Nuclear Medicine, Deyang People’s Hospital, from 2019 to 2022. All patients underwent total or subtotal thyroidectomy, followed by radioactive iodine (RAI) therapy. Patients aged ≥ 18 years who met the following criteria were included: (1) complete surgical records; (2) complete pathological records; (3) complete preoperative laboratory examinations, including TSH, FT3, FT4, antithyroglobulin antibody (TgAb), and thyroid peroxidase antibody (TPOAb); and (4) no use of thyroid‐related drugs. Patients lost to follow‐up, those with a follow‐up time of less than 1 year, and those with distant metastases at initial diagnosis were excluded. This retrospective study was approved by the Ethics Committee of Deyang People’s Hospital (2023‐04‐075‐K01), and informed consent was waived for all patients due to its retrospective nature.

### 2.2. Data Collection

The following information was collected from the patients: sex, age, height, weight, tumor staging, number of lymph node (LN) metastases, number of dissected LNs, risk stratification for recurrence, as well as preoperative thyroid function indicators and TgAb and TPOAb. The detection of thyroid function and antibodies was performed using electrochemiluminescence immunoassay (Roche, Cobas e601), and all patients were tested using the same method in the same laboratory. The reference ranges for TSH, FT3, and FT4 are 0.27–4.2 mU/L, 3.1–6.8 pmmol/L, and 12–22 pmol/L, respectively. The normal range for TgAb is below 115 IU/mL, and for TPOAb, it is below 34 IU/mL.

### 2.3. Follow‐Up and Assessment

Patients were treated with RAI after discontinuing LT4 and following a low‐iodine diet for 3 weeks, with TSH confirmed to be > 30mU/L. The dose of RAI was determined based on the specific disease severity of each patient. After the completion of RAI treatment, patients underwent structural TSH suppression therapy. Six months after the initial RAI treatment, patients stopped taking LT4 and were evaluated. Subsequently, patients were regularly reviewed, with tests including TSH, FT3, FT4, Tg, and TgAb. Neck ultrasound examinations were conducted every 6 months, and if structural lesions were suspected, they were performed every 3 months. A chest CT scan was conducted annually. Depending on the condition, SPECT/CT, CT, and PET/CT were performed as needed. Referring to the 2015 ATA guidelines [7], dynamic evaluation of patients was mainly based on serological indicators and imaging results according to the most recent review results of the patients. Based on the response to treatment, patients were divided into an ER group and a non‐ER group. The non‐ER group included IDR, BIR, and SIR.

### 2.4. Statistical Analysis

All statistical analyses and data visualizations were performed using R statistical software, Version 4.4.0 (R Foundation for Statistical Computing; https://www.r-project.org/), and *p* < 0.05 was defined as statistically significant. Continuous variables are expressed as mean ± standard deviation (SD), and categorical variables are expressed as percentages. Multivariate logistic regression analysis was used to calculate odds ratios (ORs) and 95% confidence intervals (CIs). Model 1 had unadjusted variables. Model 2 included the variables of Model 1 and adjusted for age, sex, and body mass index (BMI). Model 3 included the variables of Model 2 and adjusted for variables that may affect FT4 and FT3, including TSH, TgAb, and TPOAb. Model 4 further adjusted for all clinically relevant variables, including T stage, N stage, number of LN metastases, number of LNs dissected, and recurrence risk stratification, in addition to the covariates included in Model 3. Subsequently, quartiles of the FT4/FT3 ratio were defined by dividing the entire cohort into four equal groups based on the 25th, 50th, and 75th percentiles of the distribution. The median value within each quartile group was calculated and used as a continuous variable for trend analysis in the regression models. Finally, restricted cubic splines (RCSs) were used to evaluate the nonlinear relationship between the FT4/FT3 ratio and the short‐term prognosis of DTC.

## 3. Results

### 3.1. Baseline Characteristics of Patients

After screening according to the inclusion and exclusion criteria, a total of 225 DTC patients (170 females, 55 males) were included in this retrospective study. At the last follow‐up, there were 159 cases (70.7%) with ER and 66 cases (29.3%) with non‐ER. In terms of thyroid hormones, FT4 was higher in the ER group than in the non‐ER group (*p* = 0.040). In terms of tumor attributes, there was no difference in the distribution of primary foci (T stage) between the two groups. However, there were differences in N stage, number of LN metastases, and number of LN dissected (*p* < 0.05). Patients in the non‐ER group had a higher N stage, more LNs dissected, and more LN metastases (Table 1).

**Table 1:** Baseline characteristics of patients.

	All *N* = 225	ER *N* = 159	Non‐ER *N* = 66	*p* value
Age	43.3 ± 12.8	43.4 ± 11.6	43.0 ± 15.3	0.870
Sex:				0.641
Female	170 (75.6%)	122 (76.7%)	48 (72.7%)	
Male	55 (24.4%)	37 (23.3%)	18 (27.3%)	
BMI	23.6 ± 3.55	24.0 ± 3.63	22.6 ± 3.19	0.008
TSH	3.01 ± 2.35	2.94 ± 2.10	3.19 ± 2.89	0.521
FT3	4.96 ± 0.80	4.95 ± 0.73	4.97 ± 0.95	0.895
FT4	16.6 ± 2.87	16.8 ± 2.77	15.9 ± 3.03	0.040
TgAb	153 ± 397	154 ± 404	150 ± 380	0.941
TPOAb	49.5 ± 91.2	44.4 ± 82.6	61.6 ± 109	0.252
LN metastasis	5.96 ± 5.95	4.83 ± 5.35	8.68 ± 6.49	< 0.001
T stage:				0.294
T1∼T2	143 (63.6%)	105 (66.0%)	38 (57.6%)	
T3∼T4	82 (36.4%)	54 (34.0%)	28 (42.4%)	
N stage:				< 0.001
N0	21 (9.33%)	21 (13.2%)	0 (0.00%)	
N1a	123 (54.7%)	95 (59.7%)	28 (42.4%)	
N1b	81 (36.0%)	43 (27.0%)	38 (57.6%)	
Number of LN dissected	19.7 (14.9)	18.2 (14.8)	23.2 (14.7)	0.021
Risk stratification of recurrent:				0.001
Low	35 (15.6%)	33 (20.8%)	2 (3.03%)	
Mediate	108 (48.0%)	77 (48.4%)	31 (47.0%)	
High	82 (36.4%)	49 (30.8%)	33 (50.0%)	

Abbreviations: BMI, body mass index; LN, lymph node; TgAb, antithyroglobulin antibody; TPOAb, thyroid peroxidase antibody; TSH, thyroid‐stimulating hormone.

### 3.2. Association Between FT4/FT3 Ratio and ER

**Figure 1:**
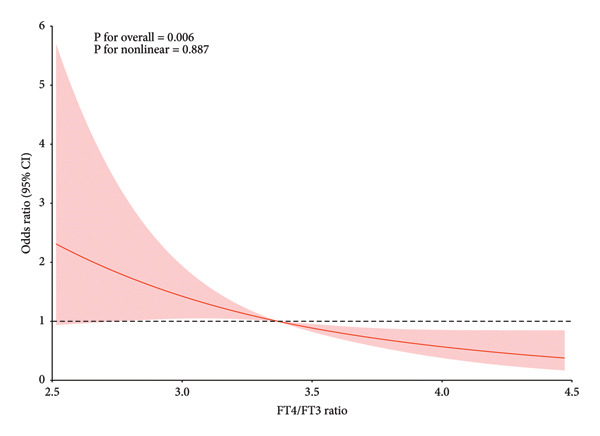
Multivariable‐adjusted restricted cubic spline curves of ER and FT4/FT3 levels in differentiated thyroid cancer assessed by a spline regression model.

To explore the association between FT4/FT3 ratio and DTC prognosis, we constructed multiple models using logistics (Table 2). After adjustment for multimodel and multivariate, the FT4/FT3 ratio was consistently associated with DTC prognosis. The OR = 0.40 and 95% CI: 0.22–0.68 after adjusting all variables. We also performed trend analysis to explore the correlation trend of the FT4/FT3 ratio with DTC prognosis (Table 3). After adjusting all covariates, we found that patients in the highest quartile of FT4/FT3 ratio (Q4, FT4/FT3 ratio > 3.74) were more likely to achieve ER status (Model 4, OR: 0.20, 95% CI: 0.07–0.55, P for trend = 0.006). Figure 1 shows the evaluation results of RCSs, and there was a dose–response relationship between the FT4/FT3 ratio and the short‐term prognosis of DTC in the safety‐adjusted model (P for overall = 0.006, P for nonlinear = 0.887).

**Table 2:** Multivariate logistic regression models for outcome.

	FT4/FT3 ratio	*p* value
	OR (95% CI)	
Model 1	0.62 (0.38–0.97)	0.047
Model 2	0.58 (0.34–0.93)	0.032
Model 3	0.58 (0.34–0.95)	0.039
Model 4	0.40 (0.22–0.68)	0.001

*Note:* Model 1: Crude. Model 2: Adjusted for Model 1 + age, sex, and BMI. Model 3: Adjusted for Model 2 + TSH, TgAb, and TPOAb. Model 4: Adjusted for Model 3 + Tstage, N stage, LN metastasis, number of LN dissected, and risk stratiBcation of recurrent.

**Table 3:** Association between FT4/FT3 ratio quartiles and ER in differentiated thyroid cancer.

Variable	OR (95% CI)				
FT4/FT3 ratio	Q1	Q2	Q3	Q4	P for trend^1^
Median^a^	2.67	3.17	3.5	4.04	
Model 1	Reference	0.54(0.24,1.18)	0.64(0.29,1.39)	0.36(0.15,0.82)	0.032
Model 2	Reference	0.51(0.22,1.14)	0.54(0.23,1.20)	0.33(0.13,0.77)	0.017
Model 3	Reference	0.52(0.22,1.16)	0.54(0.24,1.22)	0.34(0.13,0.80)	0.023
Model 4	Reference	0.51(0.19,1.27)	0.59(0.22,1.52)	0.20(0.07,0.55)	0.006

^a^Trend analysis based on variables that include the median of each quartile.

^1^Test for trend based on variable containing median value for each quartile.

## 4. Discussion

This study investigated the relationship between preoperative baseline FT4/FT3 ratio and short‐term ER in DTC. Our results showed that patients with a higher FT4/FT3 ratio were more likely to achieve an ER status (OR = 0.40, 95% CI: 0.22–0.68), suggesting that the FT4/FT3 ratio has potential as an important predictor.

The relationship between thyroid hormones and different types of tumors has been extensively studied. Majos et al., through grouping studies on the FT3/FT4 ratio, believe that the conversion rate of peripheral thyroid hormones is an independent influencing factor affecting the prognosis of pancreatic cancer [15]. In addition, in metastatic urothelial carcinoma and advanced metastatic colorectal cancer, a high FT3/FT4 ratio is associated with better progression‐free survival (PFS) and overall survival (OS) [17, 18]. However, the study by Mario et al. differs, finding a nonlinear relationship between baseline FT3/FT4 ratio and survival rates in recurrent wild‐type IDH glioblastoma [19]. It is worth noting that all the subjects in these studies were patients with advanced tumors, and the atrophy of adipose tissue and skeletal muscle is a marker of metastatic cancer [20], which may also lead to hypothyroidism syndrome. Therefore, the lower the FT3/FT4 ratio, the worse the prognosis might be.

The FT4/FT3 ratio can comprehensively reflect the synthesis and peripheral conversion status of thyroid hormones and is a sensitive indicator for measuring deiodinase activity and tissue‐level hormone sensitivity [21]. A study of 1998 patients undergoing surgery for thyroid nodules found an inverse association between peripheral sensitivity to thyroid hormone and papillary thyroid carcinoma, with the OR of 0.18 (95% CI: 0.03–0.96) for each SD increase in the ratio of FT3/FT4, reflecting the potential value of the FT4/FT3 ratio in the diagnosis of thyroid tumors [22]. In the field of thyroid cancer, more attention is paid to whether it is in ER for a long time rather than OS because of its good therapeutic effect. Studies have shown that peripheral hormone conversion, deiodinase activity, and hormone sensitivity indicators have independent predictive effects on DTC risk and treatment response [23]. When DTC patients are treated with tyrosine kinase inhibitors (TKIs), it is found that the TKI‐related increase in TSH is associated with a decrease in FT3 and FT3/FT4 ratio, and increasing the dose of LT4 alone cannot fully compensate in some patients [24]. Liu et al. found that high expression of FT3/FT4 was associated with a low recurrence rate [25]. This is different from our study results, and the possible reason is that we set different research endpoints. FT3 is negatively correlated with inflammatory status [26], and RAI treatment will cause radioactive inflammation on residual thyroid tissue or tumor tissue, and a slow level of FT3 may enhance the effect of RAI treatment. Patients with only low FT3 levels showed better survival rates than patients with both low FT3 and FT4 levels [26]. In view of this consideration, although our study found no nonlinear relationship between the FT4/FT3 ratio and the short‐term prognosis of DTC (P for nonlinear = 0.887), there may be a “U” shaped relationship between the FT4/FT3 ratio and the prognosis of thyroid cancer from the overall prognosis point of view, which needs more studies to prove.

Our study found a dose–response relationship between the FT4/FT3 ratio and the short‐term prognosis of DTC, further reinforcing the potential biological link between the two. Trend analysis (Table 3) showed that as the quartile of the ratio increased (from Q1 to Q4), the probability of patients achieving ER exhibited a significant increasing trend (P for trend < 0.001). Specifically, compared to the lowest quartile group, the adjusted OR of achieving ER in the highest quartile group was 0.20 (OR: 0.20, 95% CI: 0.07–0.55). RCS analysis (Figure 1) further confirmed that this association presented a linear dose–response relationship. The existence of this relationship suggests that the FT4/FT3 ratio may not only be a simple prognostic marker but also participate in the pathophysiological process of DTC. For instance, studies have found that thyroid hormones can regulate multiple signaling pathways related to tumor progression, such as PI3K/AKT and MAPK pathways [27]. Changes in the FT4/FT3 ratio may affect the activity of these pathways, thereby impacting the progression and prognosis of DTC. Moreover, thyroid hormones activate endothelial cell proliferation and migration through integrin αvβ3 receptors, promoting the formation of new blood vessels. This mechanism involves the interaction of multiple signaling pathways, such as NF‐κB and ERK phosphorylation, which ultimately lead to the transcriptional activation of angiogenesis‐related genes [28, 29]. The FT4/FT3 ratio not only reflects the level of thyroid hormones but may also be associated with the tumor microenvironment and immune status [15, 30], all of which could potentially influence the treatment response and prognosis of DTC patients.

However, we must interpret these results with caution. First, as a retrospective study, there may be selection bias and information bias, which cannot demonstrate causality. Second, although we adjusted for multiple potential confounders, there may still be unidentified confounders. For example, the status of iodine intake may affect thyroid hormone levels [31], but this factor was not assessed in our study. In addition, there may still be unmeasured or unadjusted chronic disease conditions (such as hepatic and renal insufficiency, among others) that introduce residual confounding to the results. Finally, our study focused on short‐term prognosis, and whether there is a “U” shaped relationship between the FT4/FT3 ratio and DTC prognosis requires a longer follow‐up time to prove. Despite these limitations, our study still provides a new perspective on understanding the prognostic factors of DTC. The FT4/FT3 ratio, as a simple and readily available indicator, may become a useful tool for assessing the prognosis of DTC.

## 5. Conclusion

Overall, our study provides preliminary evidence for the relationship between the FT4/FT3 ratio and short‐term prognosis in DTC. This finding not only helps to improve the prognostic assessment of DTC but also offers new insights into further research on the role of thyroid hormones in the development of DTC. In the future, more prospective studies and mechanistic research are needed to validate and deepen our findings, with the ultimate goal of improving the clinical management and prognosis of patients with DTC.

## Conflicts of Interest

The authors declare no conflicts of interest.

## Funding

The authors received no specific funding for this work.

## Data Availability

Research data are not shared.
